# Cold plasma effect on the proteome of *Pseudomonas aeruginosa* – Role for bacterioferritin

**DOI:** 10.1371/journal.pone.0206530

**Published:** 2018-10-26

**Authors:** Ka Pui Sharon Yau, Anthony B. Murphy, Ling Zhong, Anne Mai-Prochnow

**Affiliations:** 1 Commonwealth Scientific and Industrial Research Organisation (CSIRO) Manufacturing, Sydney, New South Wales, Australia; 2 School of Biotechnology and Biomolecular Sciences, University of New South Wales, Sydney, New South Wales, Australia; 3 Bioanalytical Mass Spectrometry Facility, Mark Wainwright Analytical Centre, University of New South Wales, Sydney, New South Wales, Australia; Kwangwoon University, REPUBLIC OF KOREA

## Abstract

Cold atmospheric-pressure plasma (CAP) is a relatively new method used for bacterial inactivation. CAP is ionized gas that can be generated by applying an electric current to air or a feeding gas. It contains reactive species and emits UV radiation, which have antibacterial activity. Previous data suggests that CAP is effective in microbial inactivation and can decontaminate and sterilize surfaces, but its exact mode of action is still under debate. This study demonstrates the effect of CAP on the whole proteome of *Pseudomonas aeruginosa* PAO1 biofilms, which is a dominant pathogen in cystic fibrosis and medical device-related infections. Liquid chromatography-mass spectrometry (LC-MS) was used to identify differentially regulated proteins of whole cell *P*. *aeruginosa* extracts. A total of 16 proteins were identified to be affected by plasma treatment compared to the control. Eight of the identified proteins have functions in transcription and translation and their expression changes are likely to be part of a general physiological response instead of a CAP-specific adaptation. However, CAP also affected bacterioferritin (Bfr), Isocitrate dehydrogenase (Idh), Trigger factor (Tig) and a chemotaxis protein, which may be involved in *P*. *aeruginosa*’s specific response to CAP. We confirm that bacterioferritin B plays a role in the bacterial response to CAP because Δ*bfrB* mutants of both PAO1 and PA14 are more susceptible to plasma-induced cell-death than their corresponding wild-type strains. To our knowledge, this is the first study showing the effect of plasma on the whole proteome of a pathogenic microorganism. It will help our understanding of the mode of action of CAP-mediated bacterial inactivation and thus support a safe and effective routine use of CAP in clinical and industrial settings.

## Introduction

Over the last century, the widespread use of antibiotics has quickly given rise to multi-drug resistant bacteria [1]. The Centre of Disease Control estimates 2 million people succumb each year to antibiotic-resistant bacterial infections, resulting in 23,000 deaths, and has suggested inappropriate prescription and use of antibiotics are major contributors to the increase in resistance [2]. Frequent use of antibacterial treatments places bacteria under biological pressure, which results in genetic alterations that subsequently improve its survival against antibiotics.

*Pseudomonas aeruginosa* is an opportunistic pathogen with an intrinsically high antibiotic resistance. Cystic fibrosis (CF) patients suffer from chronic bacterial pulmonary infections with *P*. *aeruginosa* as the predominant respiratory pathogen [3]. The recurring infections have been proven to be difficult to treat with current antibiotic regimens; this has been thought to be due to the formation of *P*. *aeruginosa* biofilms in the CF patient’s airways [3]. In addition to secretion of actin and extracellular DNA from the extracellular polymeric substance (EPS), formation of biofilms in the airways produce an excess amount of mucous that coats the airway, creating a low oxygen environment ideal for enhanced bacterial growth [4].

Biofilm formation is a characteristic microbial development that was shown to increase antibiotic resistance in microorganisms [4, 5]. Biofilms are a group of microbial cells enclosed in a matrix of EPS that are attached to a surface [4]. This specific structure serves to cement the whole biofilm community, instead of individual cells, to a surface [5]. It was demonstrated that biofilm cells show a higher resistance to antimicrobial methods due to a range of differences from their planktonic counterparts, including the presence of the EPS, oxidative stress response, differential gene or protein expression, and the presence of persister cells [6–8]. A higher resistance means that biofilm killing often needs longer treatment times than killing of planktonic cell cultures of the same species in a direct comparison [9–11].

Cold atmospheric-pressure plasma (CAP) has been investigated for its anti-bacterial, -fungal and -viral properties in *in vitro* models [11–13]. CAP is partially ionized gas created by applying high voltage electricity to a gas at atmospheric pressure. The electrons are at high temperature, while the heavy species (molecules, atoms and ions) remain close to room temperature. CAP contains reactive species including excited molecules radicals and ions, and emits UV radiation [14, 15]. Depending on operating conditions and the choice of gas, a mixture of reactive oxygen and nitrogen species (RONS) is produced that has antibacterial effects. Some studies have included high oxygen content gases in combination with atmospheric oxygen and nitrogen species, which introduces a higher diversity of RONS e.g. hydrogen peroxide (H_2_O_2_), nitric oxide (NO) and peroxinitrate (O_2_NOO^-^) [14–16]. Along with the above plasma components, electrons and neutral atoms target cellular and metabolic processes in microorganisms that can cause oxidative stress resulting in cell damage [17, 18]. Bacteria have developed several mechanisms of regulating oxidative stress to increase cell survival under these conditions. This includes the increased expression of *P*. *aeruginosa* MexXY-OprM multidrug efflux system, which is commonly associated with aminoglycoside resistance in cystic fibrosis isolates, following increased peroxide exposure [19, 20].

Several CAP studies have been conducted on well-known human pathogens such as *P*. *aeruginosa*, *Escherichia coli* and several *Staphylococcus* spp. Application of plasma to bacterial cells has proven effective in microbial inactivation, decontamination and sterilization of surfaces and wound healing [11, 21–23]. However, only limited data regarding the mechanism of CAP-induced cell death is available and in particular the effects on bacterial genomes and/or proteomes remain to be elucidated.

In this study, we investigated the effect of CAP treatment on the proteome of *P*. *aeruginosa* biofilm cells. We identified 16 differentially regulated proteins following CAP treatment using LC-MS/MS. Eight proteins involved in ribosomal machinery were found to be upregulated following plasma treatment. Interestingly, bacterioferritin Bfr was also highly expressed after 3 min plasma exposure. Further studies into the role of Bfr in biofilm survival upon CAP treatment showed that a Δ*bfr* transposon mutant strain was more susceptible to CAP treatment than its wild-type counterpart, suggesting a role for ferritin in CAP-induced cell death, possibly due to oxidative stress.

## Experimental procedure

### Bacterial strains and culture conditions

*P*. *aeruginosa* PAO1 and PW6979 BfrB-C04:: ISlacZ/hah were obtained from PAO1 transposon mutant library [24] and strains *P*. *aeruginosa* PA14 wildtype and Δ*bfrB* mutant were obtained from http://pa14.mgh.harvard.edu/cgi-bin/pa14/home.cgi [25].

Bacteria were cultivated on nutrient agar (1g l^-1^ `Lab-Lemco’ powder, 2g l^-1^ 170 yeast extract, 5g l^-1^ peptone, 5g l^-1^ sodium chloride, 15g l^-1^ agar, pH 7.4; Oxoid) using standard methods. Overnight cultures were inoculated into 10 ml nutrient broth and incubated at 37°C with 150 rpm shaking.

### Biofilm formation

Biofilms were grown in a **C**entre for Disease Control **B**iofilm **R**eactor (CBR, Biosurface Technologies, Bozeman, MT, USA) according to the standard protocol [26]. Briefly, bacteria were inoculated with 10 ml of overnight culture in tryptic soya broth (20g l^-1^ ‘Tryptic Soya Broth’ powder; Oxoid; TSB) grown at 37°C in a shaking incubator. Samples were grown on stainless steel coupons in a bioreactor containing 500 ml of TSB (600 mg l^-1^), the bioreactor was then switched to continuous-flow (11.7 ml min^-1^) with fresh TSB (100 mg l^-1^) media once cells had attached to the coupons. After overnight growth, the coupons were aseptically removed from the encasing rods and placed into 24-well plates. Coupons were rinsed twice with 1 ml phosphate buffer saline (PBS) solution to remove non-adhered cells.

### Cold atmospheric-pressure plasma (CAP) treatment

Plasma treatment was performed using the kINPen med (Neoplas tools GmbH, INP Greifswald, Germany) as previously described [11]. Briefly, coupons were placed 1 cm away from the plasma and the kINPen was operated with argon at 3.2 slm. Plasma treatment was conducted in triplicates for two time-points, 3 or 10 min, respectively. As a control, coupons were treated with non-ionized argon gas for 10 min at 2.8 slm; the plasma does not ignite for flow rates below 3.0 slm.

After treatment, coupons were submerged in 1 ml PBS solution to rehydrate surviving cells. Biofilms were scraped from the coupon surface using a flat-edge spatula. In addition, coupons and all liquids were sonicated for 5 min to dissolve possible cell clumps and encourage remaining biofilm to detach from coupon surface. The removal of cells from coupons was confirmed by microscopy and plate count (data not shown).

### Preparation of whole cell samples

Samples derived from plasma-treated biofilms were processed at the Bioanalytical Mass Spectrometry Facility (BMSF) at the University of New South Wales. Biofilm samples for LC-MS/MS were stored as pellets pooled from 24 coupons. Samples were centrifuged in an Eppendorf MiniSpin centrifuge (12,000 × g, 3 min). The cell pellets were washed once in PBS and then frozen at -20°C. Cells were lysed using 40 μl of 2% sodium dooxycholate (SDC), 1 μl 100 mM dithiothreitol (DTT) and 1 μl protease inhibitor (MMSAFE) followed by 30 min of sonication (Unisonics). Cellular debris was removed by centrifugation in an Eppendorf MiniSpin centrifuge (12,100 x g, 5 min) and the supernatant discarded.

### Quantification and in-solution digestion of proteins

The protein concentration in the lysates was measured using a 2-D quantification kit as per the manufacturer’s instructions (GE Healthcare Life Science). Briefly, proteins were incubated at 37°C in reduction (1 **μ**l 5mM DTT) and alkaline (2 **μ**l 5mM iodoacetamide) buffers for 15 and 20 min, respectively. Residue SDC was removed by the addition of 10 **μ**l 10 mM ammonium bicarbonate, 5 **μ**l Millipore water and further extracted using 2.5 **μ**l 10% trifluoroacetic acid (TFA) and centrifuged in an Eppendorf MiniSpin centrifuge (12,100 × g, 10 min). Finally, the peptides were desalted and concentrated using a StageTips^TM^ C18 microcolumn according to the manufacturer’s instructions (Thermo Scientific).

### Liquid chromatography-mass spectrometry (LC-MS/MS)

Two-point-five **μ**l of reconstituted, digested peptides were separated by nanoLC using an Ultimate nanoRSLC UPLC and autosampler system (Dionex, Amsterdam, Netherlands). Peptides were eluted using a linear gradient of H_2_O:CH_3_CN (98:2 volume, 0.1% formic acid) to H_2_O:CH_3_CN (64:36 volume, 0.1% formic acid) at 500 nl min^-1^ over 30 min. High voltage (2000 V) was applied to a low-volume titanium union (Valco) with the column oven heated to 45°C (Sonation, Biberach, Germany) and the tip positioned ~0.5 cm from the heated capillary (*T* = 300°C) of a QExactive Plus (Thermo Electron, Bremen, Germany) Mass Spectrometer. Positive ions were generated by electrospray and the QExactive operated in data-dependent acquisition mode. A survey scan of mass-to-charge ratio 350–1750 was acquired (resolution = 70,000 at m/z 200, with an accumulation target value of 1,000,000 ions) and lockmass enabled (m/z 445.12003). Up to the 10 most abundant ions (> 80,000 counts, underfill ratio 10%) with charge states > +2 and < +7 were sequentially isolated (width *m*/*z* 2.5) and fragmented by higher-energy collisional dissociation (normalized collision energy = 30) with an automatic gain control target of 100,000 ions (resolution = 17,500 at m/z 200). *M*/*z* ratios selected for MS/MS were dynamically excluded for 30 seconds.

### Initial data validation and analysis

Peak lists were generated using Mascot Daemon/Mascot Distiller (Matrix Science, London, England) using default parameters, and submitted to the database search program Mascot (version 2.5.1, Matrix Science). Search parameters: precursor tolerance 4 parts per million (ppm) and product ion tolerances ± 0.05 Da; Met(O) carboxyamidomethyl-Cys specified as variable modification, enzyme specificity was trypsin, 1 missed cleavage was possible and the non-redundant protein *Pseudomonas* database from NCBI (Jan 2015) searched. Peptide and protein identifications generated from the peaks were validating using Scaffold (version 4.6.1, Proteome Software Inc., Portland, OR). Peptide identifications were accepted if they could be established at greater than 95.0% probability using the Scaffold delta-mass correction. Protein identifications were accepted if they could be established at greater than 99.0% probability and contained at least 2 identified peptides. Statistical analysis of proteins detected at each plasma time point was completed using the t-test (p < 0.1) in addition to significance in fold-change to compare changes in protein levels across samples. Functional information proteins of significance were gathered from the Pseudomonas Genome Database [27]. The basic alignment search tool (BLAST) was used to search for sequence similarities to hypothetical or unknown proteins using UniProt accession numbers and protein sequences database [28]. Protein–protein interaction networks were built using the Search Tool for the Retrieval of Interacting Genes/Proteins (STRING, v10) with a medium confidence level (0.4) and all available predication methods [29].

### CAP treatment of *P*. *aeruginosa* PAO1 and PA14 *ΔbfrB* mutants

To further investigate upregulation of bacterioferritin following CAP treatment, the susceptibility to plasma treatment of PAO1 and PA14 wild-type strains and their respective *ΔbfrB* mutants was measured to examine potential differences between strains. Biofilms were allowed to form on stainless steel coupons placed in 24 well plates with nutrient broth for 48 h. Coupons were aseptically removed and washed twice with PBS before plasma treatment was performed and cells removed from the coupon as described above. Cells were serial diluted in PBS and plated onto nutrient agar. Plates were incubated overnight at 37°C before counting colony forming units.

## Results and discussion

**Fig 1:**
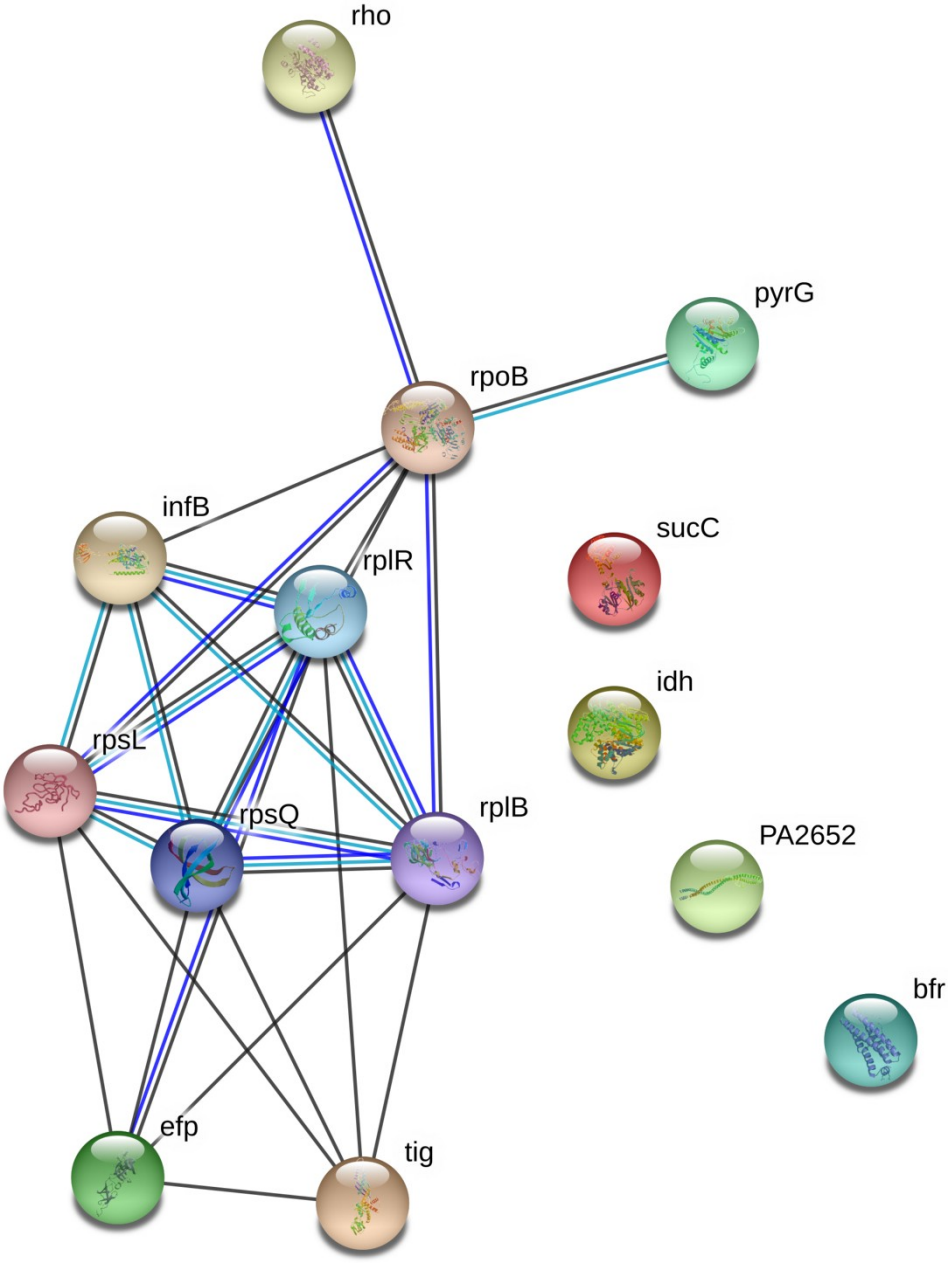
Protein interactions for unique and significantly increased proteins in plasma-treated *P*. *aeruginosa* biofilms. Interactions were detected by STRING (29). Lines indicate known or predicted protein–protein interactions. Black lines indicate proteins that are co-expressed, green lines indicate proteins within the same gene neighbourhood, blue lines indicate proteins that may be functionally linked based on gene co-occurrence. These networks show the interactions between proteins associated with metabolic processing.

**Fig 2:**
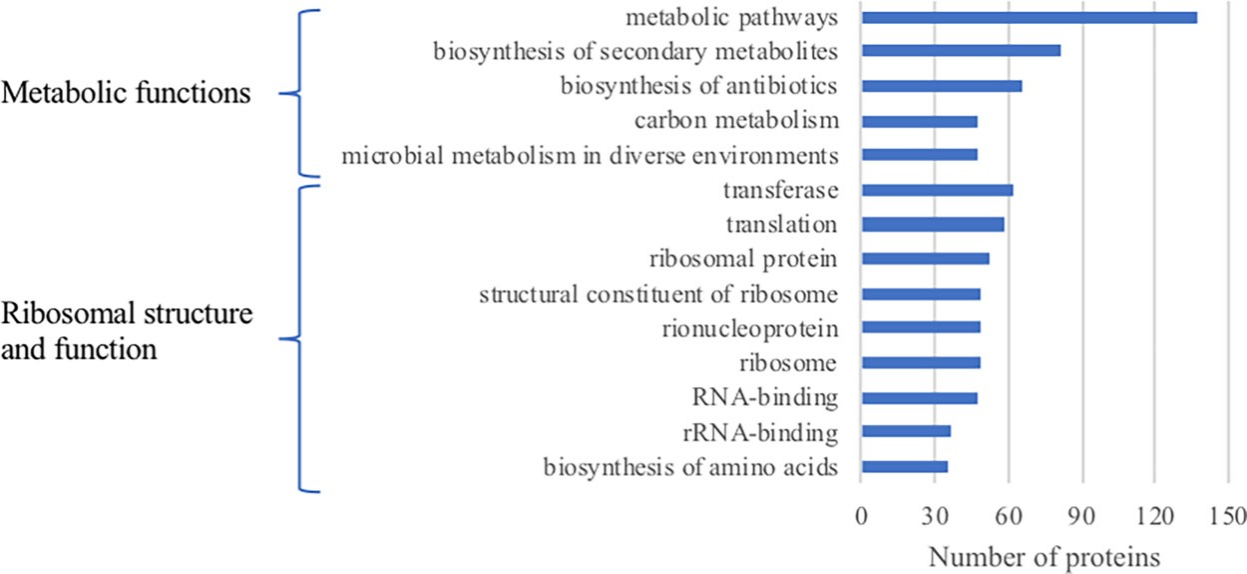
Functional annotation by DAVID Bioinformatics Resources 6.8 of proteins upregulated in plasma-treated biofilm samples. Functional groups are listed if the proteins they contain exceed 10% of total number of identified proteins.

From Scaffold, a total of 16 proteins were identified to be of significance (p > 0.1) in total spectrum counts and fold change between plasma treatment duration (Table 1). Eight out of 16 of the identified proteins have functions in transcription and translation, including RplR, RplB, RpsL, Rho, Efp, RpsQ, RpoB and InfB. Two of the identified proteins (SucC, Idh) are involved in the energy metabolism of the cell, one protein is a chemotaxis protein and one protein (PyrG) is important for nucleotide biosynthesis. Interestingly, two proteins, trigger factor (Tig) and bacterioferritin (BfrB), are important for adaptation and protection of the cell. STRING was used to identify protein–protein interaction networks present in plasma-treated samples, as depicted in Fig 1. The protein interaction networks indicated extensive connections between proteins that were associated with genomic and metabolic processes, and in the removal of reactive species. Additionally, a functional annotation analysis using Database for Annotation, Visualization, and Integrated Discovery (DAVID v6.8) was also conducted (Fig 2) on all proteins identified (S1 Table). This shows that out of 317 identified proteins, 137 (42%) have roles in metabolic pathways. A table listing all 317 proteins identified from the LC-MS/MS data is provided in the supplementary material (S1 Table).

**Table 1:** Proteins found to be upregulated following plasma treatment; p < 0.1.

Gene no.	Gene name		Function	Fold-change	Localization	Function
**Control vs 10 min CAP treatment**						
PA0859	AruC/ArgD	N2-Succinylornithine 5-aminotransferase (SOAT) = N2-acetylornithine 5-Aminotransferase (ACOAT)		>4.3	Unknown	Hypothetical
PA4247	RplR	50S ribosomal protein L18		7.3	Cytoplasmic	Translation, post-translational modification, degradation
PA4260	RplB	50S ribosomal protein L2		5.3	Cytoplasmic	Translation, post-translational modification, degradation
PA4268	RpsL	30S ribosomal protein S12		>4.3	Cytoplasmic	Translation, post-translational modification, degradation
PA5239	Rho	Transcription termination factor Rho		2.6	Cytoplasmic membrane,	Transcription, RNA processing & degradation
**Control vs 3 min CAP treatment**						
PA1588	SucC	Succinyl-CoA synthetase beta chain		6.5	Cytoplasmic	Energy metabolism
PA1800	Tig	Trigger factor		>4.67	Cytoplasmic, Outer Membrane Vesicle	Cell division, chaperones & heat shock
PA2624	Idh	Isocitrate dehydrogenase		>4.67	Unknown (multiple localization sites)	Energy metabolism
PA2652	-	Methyl-accepting chemotaxis protein		5.9	Cytoplasmic Membrane	Chemotaxis
PA2851	Efp	Translation elongation factor P		>7.5	Cytoplasmic	Translation, post-translational modification, degradation
PA3531	Bfr	Bacterioferritin		>3.3	Cytoplasmic, Outer Membrane Vesicle	Adaptation, protection, transport of small molecules
PA3637	PyrG	CTP synthase		>4	Cytoplasmic	Nucleotide biosynthesis and metabolism
PA4254	RpsQ	30S ribosomal protein S17		>5.3	Cytoplasmic	Translation, post-translational modification, degradation
PA4260	RplB	50S ribosomal protein L2		11	Cytoplasmic	Translation, post-translational modification, degradation
PA4270	RpoB	DNA-directed RNA polymerase beta chain		3.5	Cytoplasmic, Outer Membrane Vesicle	Transcription, RNA processing & degradation
PA4744	InfB	Translation initiation factor IF-2		>7.7	Cytoplasmic	Translation, post-translational modification, degradation

The highest number of proteins that were found to be upregulated after plasma treatment are involved in transcription and translation processes and are ribosomal proteins (Table 1). Changes in the expression level of these proteins are unlikely to be a specific response to CAP. Instead, the changes could be a reflection of general metabolic shift and changes in cell physiology due to stress. Additionally, the very high abundance of ribosomal proteins in the bacterial cell making a significant change in expression levels more likely due to higher change of random variations occurring for larger samples.

Two of the proteins upregulated after CAP treatment are succinyl-CoA synthetase (SucC) and isocitrate dehydrogenase (Idh). Both proteins have functions in the energy metabolism in the cell. Changes in expression of proteins that are involved in energy metabolism have been observed as a response to antibiotic stress, in particular when a subpopulation of cells become persister cells [30]. In *P*. *aeruginosa*, SucC, as part of the *sucCD* operon, preferentially synthesizes ATP and GTP, but is also capable of generating UTP or CTP. It was shown that the resulting GTP can serve as an alternative source for alginate, an important exopolysaccharide for biofilm formation, as well as for the synthesis of other macromolecules requiring GTP such as RNA and protein [31]. It could be speculated that changes in energy metabolism protein levels after CAP treatment may indicate a response of the cell to ensure survival of a subpopulation of cells.

Interestingly, two of the upregulated proteins can be categorized as having adaptation and protection functionality. One of the proteins is trigger factor (Tig). The Tig protein, is together with DnaK, involved in folding of newly synthesized proteins, and it has been shown that cells without Tig and DnaK are not viable above 30°C [32]. The other protein is bacterioferritin (BfrB). The possible involvement in protection from CAP-induced oxidative stress regulation is discussed below.

### Bacterioferritin is upregulated after CAP treatment

Bacterioferritin B (BfrB) was highly upregulated after CAP treatment (Table 1). This family of proteins has functions in adaptation, protection or transport of small molecules. Bacterioferritins are important for the regulation of the intracellular iron concentration and were shown to play a role in regulating an oxidative stress response via the iron metabolism.

In prokaryotes, iron is an important cofactor of many enzymatic reactions necessary for survival. Soluble iron (Fe^2+^) is moderately required for respiration but, due to incomplete O_2_ reduction under aerobic conditions, it produces a range of toxic reactive oxygen species [33, 34]. Ferritins and bacterioferritins (Bfr) are iron storage proteins that *P*. *aeruginosa* procures to store iron and regulate intracellular iron concentration. In *P*. *aeruginosa*, two different types of bacterioferritin exist: bacterioferritin-A (with α-subunit) and bacterioferritin-B (with a β-subunit) [35]. These molecules oxidize Fe^2+^ into Fe^3+^. The iron is stored in the form of ferrihydrite or ferric phosphate, depending on the presence of phosphate, and released under iron-limiting conditions. *P*. *aeruginosa* also makes use of DNA-binding proteins that protect the chromosome from iron-induced hydroxyl damage [36]. Bacterioferritins consists of a ferroxidase centre made up of histidine and glutamine acids that binds and oxidizes Fe^2+^ to Fe^3+^, which is then stored in the central cavity. Superoxides have been observed to mobilize the stored iron to its reactive Fe^2+^ state and cause oxidative damage. Other reports suggest that hydrogen peroxide and superoxide anions may activate the iron regulator protein [37].

In murine models, cells treated with RONs-producing drugs have shown a 6-fold increase in ferritin production and cells exposed to iron-containing substances, e.g. haemin, were able to reduce cytotoxic responses to high doses of hydrogen peroxide [37]. Orino et al. showed that increased synthesis of ferritin occurs in HeLa cells exposed to oxidative stress and that an overexpression of ferritin reduced the accumulation of ROS in response to oxidant challenge [37]. These reports suggest the role of ferritin as a protection against reactive species. The oxidative stress response in bacteria is coordinated with iron homeostasis, as reviewed by [38]. In *P*. *aeruginosa* biofilms, the importance of exogenous iron plays a significant role in biofilm formation. This is shown by the use of the mammalian iron chelator lactoferritin, which induced continuous twitching motility and a reduced biofilm thickness [39].

**Fig 3:**
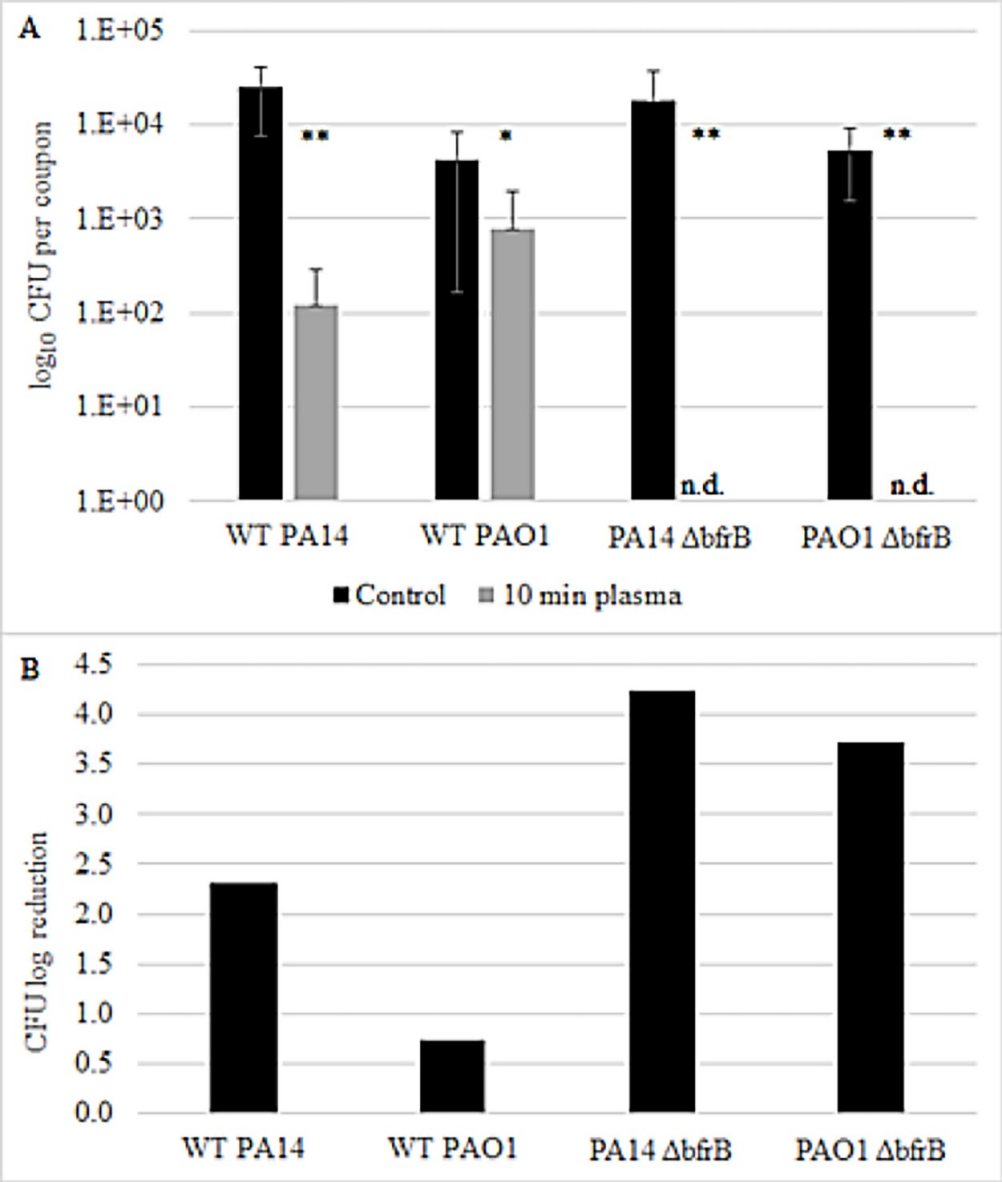
Effect of CAP treatment on cell numbers of *P*. *aeruginosa* PAO1 and PA14 wild-types and corresponding Δ*bfrB* mutants. (A) Number of log_10_ CFU recovered from biofilm coupons of control (black bar) and after 10 min CAP treatment (grey bar) for all four strains; n. d. = not detected. Values represent average of three coupons per treatment and error bars show the standard deviation. Data is shown as significant with either p < 0.05 (95% confidence, *) or p < 0.01 (99% confidence, **), based on a Student’s t-test (2 tailed, homoscedastic). (B) CFU log reduction after 10 min CAP for all four strains.

To show whether bacterioferritin plays a role in the bacterial response to cold plasma treatment, we investigated two *P*. *aeruginosa* strains (PAO1 and PA14) and their corresponding Δ*bfrB* mutants for survival after CAP treatment (Fig 3). We hypothesized that if the resulting oxidative stress from plasma treatment leads to an increase in ferritins as a protection, mutations in ferritin genes may result in lower survival of the strains upon CAP treatment. Indeed, our results show that both *P*. *aeruginosa* wild-type strains have a higher survival rate after 10 min CAP treatment compared to the Δ*bfrB* mutants (Fig 3). CAP treatment only led to a small (0.74) log reduction in CFU numbers in *P*. *aeruginosa* PAO1 wild-type (from 4.24 x 10^3^ CFU control to 7.78 x 10^2^ after plasma treatment) and 2.31 log reduction in *P*. *aeruginosa* PA14 wild-type, respectively (from 24.33 x 10^3^ CFU control to 1.2 x 10^2^ after plasma treatment). In contrast, for both *P*. *aeruginosa* PAO1 and PA14 Δ*bfrB* mutants, 10 min CAP treatment completely eliminated the biofilms with no viable cells detected after treatment (Fig 3B). These results suggest that BfrB has a protective effect for the cells against CAP-induced oxidative damage in *P*. *aeruginosa*.

Lack of functional bacterioferritins in *P*. *aeruginosa* will reduce the microorganism’s ability to provide iron required for respiration. In addition, the ability to regulate intracellular iron concentrations would be reduced in Δ*bfrB* mutants compared to the wild-type with fully functional bacterioferritin genes. Thus, it is likely that the higher cell death in the *bfrB* mutants may be due to the synergistic effects of the CAP-induced oxidative stress and the toxic compounds derived from reactive Fe^2+^.

## Conclusions

Biofilms are increasingly being recognized by the public health community as an important determinant in persistent chronic microbial infections. Additionally, biofilms can be problematic for many industries by fouling surfaces and contaminating food. CAP has the potential to eliminate biofilms that are resistant to conventional antimicrobial methods in a fast and reliable way. Our results show that the mode of action of CAP is linked to oxidative stress regulation in *P*. *aeruginosa*. We identified bacterioferritin B as one of the proteins affected by CAP and further results suggest that BfrB gives protection to *P*. *aeruginosa* upon CAP exposure. This is consistent with observations of the role of bacterioferritins in the response to oxidative stress of other organisms Identifying the mode of action of CAP in treating bacteria is an important step towards the routine use of CAP in industry and healthcare settings.

## Supporting information

S1 Table A list of proteins that are identified in plasma-treated biofilm samples from LC-MS/MS data. Proteins identified are recognised based on number of unique peptides detected across 3 biological replicates (Rep.1, Rep. 2, Rep. 3) each from 3 different treatment conditions: 10 min gas treatment, 3 min plasma treatment and 10 min plasma treatment. Percentage of average sequence coverage refers to the percentage of all the amino acids in the protein sequence that were covered by identified peptides detected in the sample. Proteins identified to be of significance (p > 0.1) in total spectrum count and fold change based upon 10 min gas treatment vs. 10 min plasma treatment and 10 min gas treatment vs. 3 min plasma treatment are presented in Table 1.
(DOCX)
