# Supplementary material for: Cold plasma effect on the proteome of Pseudomonas aeruginosa – Role for bacterioferritin
Source: PLoS One. 2018 Oct 26;13(10):e0206530. doi: 10.1371/journal.pone.0206530 (PMC6203385; doi:10.1371/journal.pone.0206530)
Supplement: S1 Table — Proteins identified are recognised based on number of unique peptides detected across 3 biological replicates (Rep.1, Rep. 2, Rep. 3) each from 3 different treatment conditions: 10 min gas treatment, 3 min plasma treatment and 10 min plasma treatment. Percentage of average sequence coverage refers to the percentage of all the amino acids in the protein sequence that were covered by identified peptides detected in the sample. Proteins identified to be of significance (p > 0.1) in total spectrum count and fold change based upon 10 min gas treatment vs. 10 min plasma treatment and 10 min gas treatment vs. 3 min plasma treatment are presented in Table 1. (DOCX) [file pone.0206530.s001.docx]

| **S1 Table. A list of proteins that are identified in plasma-treated biofilm samples from LC-MS/MS data.** Proteins identified are recognised based on number of unique peptides detected across 3 biological replicates (Rep.1, Rep. 2, Rep. 3) each from 3 different treatment conditions: 10 min gas treatment, 3 min plasma treatment and 10 min plasma treatment. Percentage of average sequence coverage refers to the percentage of all the amino acids in the protein sequence that were covered by identified peptides detected in the sample. Proteins identified to be of significance (p > 0.1) in total spectrum count and fold change based upon 10 min gas treatment vs. 10 min plasma treatment and 10 min gas treatment vs. 3 min plasma treatment are presented in Table 1. | | | | | | | | | | | | |
| --- | --- | --- | --- | --- | --- | --- | --- | --- | --- | --- | --- | --- |
| Accession number | Gene | Identified Protein | Unique peptides detected | | | | | | | | | Avg sequence coverage % |
|  |  |  | 10 min gas treatment | | | 3 min plasma treatment | | | 10 min plasma treatment | | |  |
|  |  |  | Rep. 1^[[1]](#footnote-1)^ | Rep. 2 | Rep. 3 | Rep. 1 | Rep. 2 | Rep. 3 | Rep. 1 | Rep. 2 | Rep. 3 |  |
| AAT_PSEAE | aspC | Aspartate aminotransferase | 0 | 0 | 0 | 3 | 5 | 0 | 0 | 3 | 2 | 5.08% |
| ACCA_PSEAE | accA | Acetyl-coenzyme A carboxylase carboxyl transferase subunit alpha | 0 | 0 | 0 | 3 | 0 | 0 | 0 | 0 | 0 | 1.72% |
| ACCC_PSEAE | accC | Biotin carboxylase | 0 | 0 | 2 | 9 | 10 | 0 | 0 | 2 | 8 | 10.44% |
| ACCD_PSEAE | accD | Cluster of Acetyl-coenzyme A carboxylase carboxyl transferase subunit beta | 0 | 0 | 2 | 3 | 4 | 0 | 0 | 0 | 4 | 7.82% |
| ACEA_PSEAE | PA2634 | Isocitrate lyase | 0 | 3 | 0 | 9 | 4 | 0 | 0 | 2 | 2 | 4.51% |
| ACKA_PSEAE | ackA | Acetate kinase | 0 | 0 | 0 | 3 | 0 | 0 | 0 | 0 | 0 | 0.99% |
| ACNA_PSEAE | acnA | Cluster of Aconitate hydratase A | 0 | 0 | 0 | 8 | 3 | 0 | 0 | 0 | 8 | 0.00% |
| ACNB_PSEAE | acnB | Aconitate hydratase B | 0 | 2 | 0 | 10 | 3 | 2 | 0 | 0 | 2 | 0.00% |
| ACP1_PSEAE | acpP1 | Cluster of Acyl carrier protein 1 | 0 | 5 | 0 | 2 | 3 | 0 | 2 | 0 | 2 | 15.39% |
| AK_PSEAE | lysC | Aspartokinase | 2 | 0 | 0 | 2 | 0 | 0 | 0 | 0 | 2 | 2.21% |
| ALF_PSEAE | fba | Fructose-bisphosphate aldolase | 0 | 0 | 3 | 4 | 3 | 0 | 0 | 3 | 5 | 8.80% |
| ALGC_PSEAE | algC | Phosphomannomutase/phosphoglucomutase | 0 | 0 | 0 | 3 | 2 | 0 | 0 | 0 | 3 | 3.26% |
| ALGP_PSEAE | algP | Transcriptional regulatory protein | 0 | 2 | 4 | 15 | 5 | 3 | 3 | 6 | 8 | 15.42% |
| APT_PSEAE | apt | Adenine phosphoribosyltransferase | 0 | 0 | 0 | 0 | 0 | 0 | 0 | 0 | 2 | 1.71% |
| ARCA_PSEAE | arcA | Arginine deiminase | 0 | 4 | 3 | 23 | 15 | 3 | 2 | 0 | 14 | 14.06% |
| ARCC_PSEAE | arcC | Carbamate kinase | 0 | 0 | 5 | 8 | 6 | 0 | 2 | 0 | 6 | 10.68% |
| ARGB_PSEAE | argB | Acetylglutamate kinase | 0 | 0 | 0 | 0 | 0 | 0 | 0 | 0 | 2 | 1.26% |
| ARGC_PSEAE | argC | N-acetyl-gamma-glutamyl-phosphate reductase | 0 | 0 | 0 | 0 | 0 | 0 | 0 | 0 | 3 | 1.46% |
| ARLY_PSEAE | argH | Argininosuccinate lyase | 0 | 0 | 0 | 0 | 2 | 0 | 0 | 0 | 2 | 1.10% |
| AROB_PSEAE | aroB | 3-dehydroquinate synthase | 0 | 0 | 0 | 2 | 0 | 0 | 0 | 0 | 0 | 1.33% |
| ARUC_PSEAE | aruC | Succinylornithine transaminase/acetylornithine aminotransferase | 0 | 0 | 0 | 0 | 0 | 0 | 2 | 3 | 6 | 4.84% |
| ASPA_PSEAE | aspA | Aspartate ammonia-lyase | 0 | 0 | 0 | 2 | 0 | 0 | 0 | 0 | 3 | 2.23% |
| ASPQ_PSEAE | ansB | Cluster of Glutaminase-asparaginase | 0 | 0 | 0 | 4 | 3 | 0 | 0 | 3 | 9 | 8.47% |
| ATOB_PSEAE | atoB | Acetyl-CoA acetyltransferase | 0 | 2 | 4 | 2 | 2 | 0 | 0 | 0 | 3 | 6.41% |
| ATPA_PSEAE | atpA | Cluster of ATP synthase subunit alpha | 3 | 17 | 24 | 34 | 26 | 13 | 11 | 19 | 33 | 23.68% |
| ATPB_PSEAE | atpD | Cluster of ATP synthase subunit beta | 0 | 27 | 19 | 31 | 29 | 10 | 14 | 17 | 17 | 29.82% |
| ATPD_PSEAE | atpH | ATP synthase subunit delta | 0 | 0 | 2 | 3 | 3 | 0 | 0 | 0 | 4 | 12.04% |
| ATPE_PSEAE | atpC | ATP synthase epsilon chain | 0 | 4 | 8 | 8 | 6 | 0 | 0 | 4 | 8 | 28.61% |
| ATPF_PSEAE | atpF | ATP synthase subunit b | 0 | 4 | 14 | 22 | 14 | 2 | 3 | 5 | 24 | 42.93% |
| ATPG_PSEAE | atpG | Cluster of ATP synthase gamma chain | 0 | 0 | 3 | 3 | 4 | 2 | 2 | 0 | 2 | 9.17% |
| AZUR_PSEAE | azu | Cluster of Azurin | 0 | 0 | 5 | 0 | 0 | 0 | 0 | 0 | 4 | 6.08% |
| BAMB_PSEAE | bamB | Outer membrane protein assembly factor | 0 | 0 | 0 | 5 | 2 | 0 | 0 | 0 | 9 | 8.10% |
| BAMD_PSEAE | bamD | Outer membrane protein assembly factor | 0 | 0 | 0 | 5 | 2 | 0 | 0 | 0 | 6 | 7.30% |
| BAUC_PSEAE | bauC | Putative 3-oxopropanoate dehydrogenase | 0 | 0 | 0 | 4 | 0 | 0 | 0 | 6 | 4 | 5.74% |
| BCCP_PSEAE | accB | Biotin carboxyl carrier protein of acetyl-CoA carboxylase | 0 | 0 | 4 | 4 | 0 | 0 | 0 | 0 | 0 | 7.62% |
| BFR_PSEAE | bfr | Bacterioferritin | 0 | 0 | 0 | 4 | 2 | 3 | 2 | 0 | 7 | 17.53% |
| BRAC_PSEAE | braC | Leucine-, isoleucine-, valine-, threonine-, and alanine-binding protein | 0 | 4 | 6 | 2 | 2 | 0 | 0 | 9 | 3 | 13.33% |
| CARB_PSEAE | carB | Cluster of Carbamoyl-phosphate synthase large chain | 0 | 0 | 0 | 5 | 6 | 0 | 0 | 0 | 0 | 1.52% |
| CATA_PSEAE | katA | Catalase | 0 | 0 | 0 | 3 | 0 | 0 | 0 | 0 | 6 | 3.16% |
| CCME_PSEAE | ccmE | Cytochrome c-type biogenesis protein | 0 | 0 | 0 | 0 | 2 | 0 | 0 | 0 | 3 | 4.59% |
| CCMF_PSEAE | ccmF | Cytochrome c-type biogenesis protein | 0 | 0 | 0 | 0 | 0 | 0 | 0 | 0 | 2 | 0.52% |
| CCMH_PSEAE | ccmH | Cytochrome c-type biogenesis protein | 0 | 0 | 0 | 0 | 0 | 0 | 0 | 0 | 3 | 2.66% |
| CCPR_PSEAE | ccpA | Cytochrome c551 peroxidase | 0 | 0 | 0 | 0 | 2 | 0 | 0 | 0 | 6 | 2.89% |
| CH10_PSEAE | groS | Cluster of 10 kDa chaperonin | 0 | 3 | 2 | 6 | 4 | 0 | 2 | 4 | 3 | 35.08% |
| CH60_PSEAE | groL | Cluster of 60 kDa chaperonin | 10 | 41 | 49 | 79 | 43 | 17 | 53 | 38 | 54 | 36.47% |
| CHEB1_PSEAE | cheB1 | Chemotaxis response regulator protein-glutamate methylesterase of group 1 operon | 0 | 0 | 0 | 0 | 2 | 0 | 0 | 0 | 0 | 0.85% |
| CLPB_PSEAE | clpB | Cluster of Chaperone protein | 0 | 8 | 7 | 12 | 10 | 0 | 0 | 2 | 14 | 6.60% |
| CLPP1_PSEAE | clpP1 | ATP-dependent Clp protease proteolytic subunit 1 | 0 | 0 | 0 | 0 | 0 | 0 | 0 | 0 | 2 | 1.98% |
| CLPX_PSEAE | clpX | Cluster of ATP-dependent Clp protease ATP-binding subunit | 0 | 0 | 0 | 0 | 3 | 0 | 0 | 0 | 0 | 0.73% |
| CSPA_PSEAE | cspA | Major cold shock protein | 0 | 0 | 0 | 3 | 0 | 0 | 0 | 0 | 4 | 17.07% |
| CSRA_PSEAE | csrA | Carbon storage regulator homolog | 0 | 2 | 4 | 3 | 2 | 2 | 0 | 3 | 2 | 40.62% |
| CYC4_PSEAE | cc4 | Cluster of Cytochrome c4 | 0 | 0 | 5 | 9 | 2 | 0 | 0 | 0 | 10 | 12.70% |
| CYCH_PSEAE | cycH | Cytochrome c-type biogenesis protein | 0 | 0 | 0 | 3 | 3 | 0 | 0 | 0 | 8 | 6.60% |
| DADA1_PSEAE | dadA1 | Cluster of D-amino acid dehydrogenase 1 | 0 | 0 | 0 | 3 | 0 | 0 | 0 | 0 | 2 | 1.98% |
| DAVD_PSEAE | davD | Cluster of Glutarate-semialdehyde dehydrogenase | 0 | 5 | 3 | 10 | 6 | 3 | 0 | 7 | 13 | 12.69% |
| DAVT_PSEAE | davT | 5-aminovalerate aminotransferase | 0 | 4 | 3 | 5 | 4 | 2 | 0 | 13 | 14 | 15.11% |
| DBHA_PSEAE | hupA | DNA-binding protein HU-alpha | 0 | 3 | 3 | 2 | 2 | 0 | 0 | 6 | 2 | 18.16% |
| DBHB_PSEAE | hupB | DNA-binding protein HU-beta | 0 | 7 | 9 | 5 | 8 | 0 | 6 | 7 | 14 | 58.27% |
| DCDA_PSEAE | lysA | Diaminopimelate decarboxylase | 0 | 0 | 0 | 0 | 2 | 0 | 0 | 0 | 0 | 0.80% |
| DCTA2_PSEAE | dctA2 | C4-dicarboxylate transport protein 2 | 0 | 0 | 0 | 2 | 0 | 0 | 0 | 0 | 0 | 0.82% |
| DEF_PSEAE | def | Peptide deformylase | 0 | 0 | 0 | 2 | 0 | 0 | 0 | 0 | 2 | 2.78% |
| DGTL2_PSEAE | dgt2 | Deoxyguanosinetriphosphate triphosphohydrolase-like protein | 0 | 0 | 0 | 0 | 0 | 0 | 0 | 0 | 2 | 0.70% |
| DHAS_PSEAE | asd | Aspartate-semialdehyde dehydrogenase | 0 | 0 | 0 | 0 | 0 | 0 | 0 | 0 | 6 | 2.01% |
| DHE2_PSEAE | gdhB | NAD-specific glutamate dehydrogenase | 0 | 0 | 0 | 3 | 4 | 0 | 0 | 0 | 0 | 0.56% |
| DLDH2_PSEAE | lpdG | Dihydrolipoyl dehydrogenase | 0 | 0 | 0 | 8 | 5 | 0 | 0 | 2 | 10 | 7.55% |
| DNAJ_PSEAE | dnaJ | Chaperone protein | 0 | 0 | 0 | 2 | 0 | 0 | 0 | 0 | 0 | 0.77% |
| DNAK_PSEAE | dnaK | Cluster of Chaperone protein | 13 | 28 | 23 | 23 | 16 | 16 | 20 | 17 | 26 | 20.46% |
| DPO1_PSEAE | polA | DNA polymerase I | 0 | 0 | 0 | 2 | 2 | 0 | 0 | 0 | 0 | 0.76% |
| DPO3B_PSEAE | dnaN | DNA polymerase III subunit beta | 0 | 0 | 0 | 2 | 0 | 3 | 0 | 0 | 6 | 4.15% |
| EFG1_PSEAE | fusA | Cluster of Elongation factor G 1 | 7 | 31 | 34 | 32 | 22 | 19 | 20 | 11 | 41 | 30.67% |
| EFP_PSEAE | efp | Cluster of Elongation factor P | 0 | 0 | 0 | 5 | 5 | 2 | 0 | 0 | 2 | 8.63% |
| EFTS_PSEAE | tsf | Cluster of Elongation factor Ts | 0 | 4 | 7 | 15 | 13 | 0 | 3 | 6 | 13 | 25.80% |
| EFTU_PSEAE | tufA | Cluster of Elongation factor Tu | 8 | 60 | 53 | 36 | 30 | 11 | 43 | 27 | 32 | 31.58% |
| ENO_PSEAE | eno | Cluster of Enolase | 0 | 0 | 7 | 23 | 15 | 0 | 37 | 6 | 6 | 11.65% |
| ETFA_PSEAE | etfA | Electron transfer flavoprotein subunit alpha | 0 | 0 | 4 | 3 | 3 | 0 | 0 | 3 | 7 | 14.96% |
| ETFB_PSEAE | etfB | Electron transfer flavoprotein subunit beta | 0 | 4 | 3 | 2 | 6 | 0 | 0 | 5 | 8 | 18.29% |
| ETFD_PSEAE | PA2953 | Electron transfer flavoprotein-ubiquinone oxidoreductase | 0 | 0 | 0 | 0 | 0 | 0 | 0 | 0 | 2 | 0.85% |
| EXAA_PSEAE | exaA | Quinoprotein ethanol dehydrogenase | 0 | 0 | 2 | 0 | 0 | 0 | 0 | 0 | 0 | 0.80% |
| F16PA_PSEAE | fbp | Cluster of Fructose-1,6-bisphosphatase class 1 | 0 | 0 | 2 | 6 | 0 | 4 | 0 | 2 | 10 | 8.73% |
| FABG_PSEAE | fabG | Cluster of 3-oxoacyl-[acyl-carrier-protein] reductase | 0 | 0 | 0 | 3 | 6 | 0 | 0 | 3 | 7 | 12.06% |
| FABY_PSEAE | fabY | Beta-ketoacyl-[acyl-carrier-protein] synthase | 0 | 0 | 0 | 5 | 4 | 0 | 0 | 0 | 7 | 0.00% |
| FADA_PSEAE | fadA | Cluster of 3-ketoacyl-CoA thiolase | 0 | 0 | 0 | 2 | 2 | 0 | 0 | 2 | 0 | 1.90% |
| FADB_PSEAE | fadB | Cluster of Fatty acid oxidation complex subunit alpha | 0 | 3 | 3 | 3 | 2 | 0 | 0 | 0 | 0 | 2.95% |
| FADH_PSEAE | fdhA | Glutathione-independent formaldehyde dehydrogenase | 0 | 0 | 0 | 0 | 0 | 0 | 0 | 2 | 0 | 1.28% |
| FLICB_PSEAE | fliC | B-type flagellin | 0 | 0 | 0 | 3 | 0 | 0 | 0 | 2 | 5 | 3.67% |
| FMPO_PSEAE | pilA | Fimbrial protein | 0 | 2 | 3 | 8 | 7 | 0 | 3 | 3 | 5 | 22.29% |
| FUMC2_PSEAE | fumC2 | Fumarate hydratase class II 2 | 0 | 0 | 0 | 0 | 0 | 0 | 0 | 0 | 4 | 1.49% |
| FUR_PSEAE | fur | Ferric uptake regulation protein | 0 | 0 | 0 | 0 | 0 | 0 | 0 | 0 | 4 | 2.57% |
| G6PI_PSEAE | pgi | Glucose-6-phosphate isomerase | 0 | 0 | 0 | 0 | 0 | 0 | 0 | 0 | 2 | 0.48% |
| GALU_PSEAE | galU | UTP--glucose-1-phosphate uridylyltransferase | 0 | 0 | 0 | 2 | 0 | 0 | 0 | 0 | 3 | 3.62% |
| GATA_PSEAE | gatA | Glutamyl-tRNA(Gln) amidotransferase subunit A | 0 | 0 | 0 | 0 | 0 | 0 | 0 | 0 | 2 | 0.69% |
| GATB_PSEAE | gatB | Cluster of Aspartyl/glutamyl-tRNA(Asn/Gln) amidotransferase subunit B | 2 | 0 | 2 | 2 | 4 | 0 | 0 | 0 | 5 | 4.06% |
| GATC_PSEAE | gatC | Glutamyl-tRNA(Gln) amidotransferase subunit C | 0 | 0 | 0 | 0 | 0 | 0 | 0 | 0 | 3 | 5.32% |
| GBUA_PSEAE | gbuA | Guanidinobutyrase | 0 | 0 | 0 | 0 | 2 | 0 | 0 | 0 | 3 | 3.27% |
| GCH12_PSEAE | folE2 | GTP cyclohydrolase 1 2 | 0 | 0 | 0 | 0 | 0 | 0 | 0 | 0 | 2 | 2.21% |
| GCSH2_PSEAE | gcvH2 | Glycine cleavage system H protein 2 | 0 | 0 | 2 | 3 | 0 | 0 | 0 | 0 | 2 | 6.63% |
| GCST_PSEAE | gcvT | Aminomethyltransferase | 0 | 0 | 0 | 0 | 3 | 0 | 0 | 0 | 0 | 1.30% |
| GLMS_PSEAE | glmS | Glutamine--fructose-6-phosphate aminotransferase [isomerizing] | 0 | 0 | 0 | 3 | 2 | 0 | 0 | 0 | 2 | 1.93% |
| GLMU_PSEAE | glmU | Cluster of Bifunctional protein | 0 | 0 | 0 | 3 | 0 | 0 | 0 | 0 | 7 | 4.51% |
| GLNA_PSEAE | glnA | Glutamine synthetase | 2 | 0 | 2 | 4 | 0 | 2 | 0 | 3 | 6 | 6.34% |
| GLYA3_PSEAE | glyA2 | Cluster of Serine hydroxymethyltransferase 3 | 0 | 0 | 0 | 0 | 5 | 2 | 2 | 0 | 0 | 0.00% |
| GREA_PSEAE | greA | Transcription elongation factor | 0 | 0 | 0 | 2 | 2 | 0 | 0 | 2 | 3 | 7.51% |
| GRPE_PSEAE | grpE | Protein GrpE | 0 | 0 | 3 | 3 | 3 | 0 | 0 | 0 | 5 | 14.52% |
| GSA_PSEAE | hemL | Cluster of Glutamate-1-semialdehyde 2,1-aminomutase | 0 | 0 | 0 | 3 | 0 | 0 | 0 | 0 | 0 | 1.14% |
| GSH1_PSEAE | gshA | Glutamate--cysteine ligase | 0 | 0 | 0 | 2 | 0 | 0 | 0 | 0 | 0 | 0.61% |
| GSHB_PSEAE | gshB | Glutathione synthetase | 0 | 0 | 0 | 2 | 0 | 0 | 0 | 0 | 0 | 0.91% |
| GSHR_PSEAE | gor | Glutathione reductase | 0 | 0 | 0 | 0 | 0 | 0 | 0 | 0 | 2 | 0.79% |
| GUAA_PSEAE | guaA | Cluster of GMP synthase [glutamine-hydrolyzing] | 0 | 0 | 3 | 4 | 4 | 0 | 0 | 0 | 2 | 3.54% |
| GYRA_PSEAE | gyrA | Cluster of DNA gyrase subunit A | 0 | 0 | 0 | 2 | 0 | 0 | 0 | 0 | 2 | 0.69% |
| HCP1_PSEAE | hcp1 | Protein hcp1 | 0 | 0 | 2 | 0 | 0 | 0 | 0 | 0 | 0 | 1.10% |
| HEM2_PSEAE | hemB | Delta-aminolevulinic acid dehydratase | 0 | 0 | 0 | 5 | 5 | 0 | 0 | 2 | 7 | 7.52% |
| HEM3_PSEAE | hemC | Porphobilinogen deaminase | 0 | 0 | 0 | 0 | 0 | 0 | 0 | 0 | 2 | 0.57% |
| HEM6_PSEAE | hemF | Oxygen-dependent coproporphyrinogen-III oxidase | 0 | 0 | 0 | 0 | 0 | 3 | 0 | 0 | 2 | 2.84% |
| HEMTB_PSEAE | PA1673 | Bacteriohemerythrin | 0 | 0 | 0 | 0 | 0 | 0 | 0 | 0 | 3 | 2.98% |
| HIS1_PSEAE | hisG | ATP phosphoribosyltransferase | 0 | 0 | 0 | 0 | 0 | 0 | 0 | 0 | 2 | 2.06% |
| HIS4_PSEAE | hisA | 1-(5-phosphoribosyl)-5-[(5-phosphoribosylamino)methylideneamino] imidazole-4-carboxamide isomerase | 0 | 0 | 0 | 2 | 0 | 0 | 0 | 0 | 0 | 1.36% |
| HIS7_PSEAE | hisB | Cluster of Imidazoleglycerol-phosphate dehydratase | 0 | 0 | 0 | 0 | 0 | 0 | 0 | 0 | 3 | 1.87% |
| HISX_PSEAE | hisD | Cluster of Histidinol dehydrogenase | 0 | 0 | 0 | 4 | 5 | 0 | 0 | 0 | 6 | 6.21% |
| HISZ_PSEAE | hisZ | ATP phosphoribosyltransferase regulatory subunit | 0 | 0 | 0 | 0 | 0 | 0 | 0 | 0 | 4 | 1.67% |
| HSLU_PSEAE | hslU | Cluster of ATP-dependent protease ATPase subunit | 0 | 0 | 2 | 4 | 0 | 0 | 0 | 0 | 6 | 4.38% |
| HTPG_PSEAE | htpG | Cluster of Chaperone protein | 0 | 0 | 2 | 0 | 2 | 2 | 0 | 0 | 2 | 2.91% |
| IF1_PSEAE | infA | Translation initiation factor IF-1 | 0 | 0 | 0 | 0 | 0 | 0 | 0 | 0 | 3 | 8.64% |
| IF2_PSEAE | infB | Cluster of Translation initiation factor IF-2 | 0 | 0 | 0 | 9 | 4 | 7 | 0 | 0 | 6 | 4.54% |
| IF3_PSEAE | infC | Cluster of Translation initiation factor IF-3 | 0 | 0 | 5 | 0 | 5 | 0 | 0 | 0 | 11 | 14.01% |
| ILVC_PSEAE | ilvC | Ketol-acid reductoisomerase | 0 | 0 | 0 | 2 | 0 | 2 | 0 | 0 | 5 | 4.01% |
| ILVE_PSEAE | ilvE | Branched-chain-amino-acid aminotransferase | 0 | 0 | 0 | 0 | 0 | 0 | 0 | 0 | 10 | 4.63% |
| IPYR_PSEAE | ppa | Inorganic pyrophosphatase | 0 | 0 | 3 | 2 | 0 | 0 | 0 | 0 | 2 | 6.60% |
| ISCS_PSEAE | iscS | Cysteine desulfurase | 0 | 0 | 2 | 3 | 0 | 0 | 0 | 0 | 2 | 2.50% |
| KAD_PSEAE | adk | Adenylate kinase | 0 | 0 | 0 | 3 | 2 | 0 | 0 | 3 | 2 | 7.97% |
| KDPB_PSEAE | kdpB | Potassium-transporting ATPase ATP-binding subunit | 0 | 0 | 0 | 3 | 0 | 0 | 0 | 0 | 0 | 0.00% |
| KDPC_PSEAE | kdpC | Potassium-transporting ATPase KdpC subunit | 0 | 0 | 0 | 2 | 0 | 0 | 0 | 0 | 0 | 0.00% |
| KDSB_PSEAE | kdsB | 3-deoxy-manno-octulosonate cytidylyltransferase | 0 | 0 | 0 | 0 | 2 | 0 | 0 | 0 | 2 | 2.27% |
| KGUA_PSEAE | gmk | Guanylate kinase | 0 | 0 | 0 | 3 | 0 | 0 | 0 | 0 | 4 | 4.22% |
| KPRS_PSEAE | prs | Cluster of Ribose-phosphate pyrophosphokinase | 0 | 0 | 0 | 4 | 2 | 0 | 5 | 0 | 4 | 6.17% |
| LEP_PSEAE | lepB | Signal peptidase I | 0 | 0 | 0 | 0 | 0 | 0 | 0 | 0 | 2 | 1.52% |
| LLDD_PSEAE | lldD | Cluster of L-lactate dehydrogenase | 0 | 0 | 0 | 3 | 3 | 0 | 2 | 0 | 5 | 4.78% |
| LOLD_PSEAE | lolD | Lipoprotein-releasing system ATP-binding protein | 0 | 0 | 0 | 2 | 0 | 0 | 0 | 0 | 0 | 0.73% |
| LPTD_PSEAE | lptD | Cluster of LPS-assembly protein | 0 | 0 | 0 | 6 | 3 | 0 | 0 | 2 | 0 | 2.32% |
| LPXC_PSEAE | lpxC | UDP-3-O-[3-hydroxymyristoyl] N-acetylglucosamine deacetylase | 0 | 0 | 2 | 3 | 4 | 0 | 0 | 4 | 6 | 8.50% |
| MASZ_PSEAE | glcB | Malate synthase G | 0 | 0 | 0 | 3 | 0 | 0 | 0 | 3 | 9 | 2.94% |
| METK_PSEAE | metK | Cluster of S-adenosylmethionine synthase | 0 | 0 | 0 | 2 | 2 | 0 | 0 | 4 | 6 | 4.35% |
| METZ_PSEAE | metZ | O-succinylhomoserine sulfhydrylase | 0 | 0 | 0 | 0 | 0 | 0 | 0 | 2 | 2 | 1.98% |
| MEXA_PSEAE | mexA | Cluster of Multidrug resistance protein | 0 | 0 | 0 | 2 | 2 | 0 | 0 | 0 | 3 | 3.16% |
| MINE_PSEAE | minE | Cell division topological specificity factor | 0 | 0 | 0 | 0 | 0 | 0 | 0 | 0 | 3 | 3.18% |
| MNMC_PSEAE | mnmC | tRNA 5-methylaminomethyl-2-thiouridine biosynthesis bifunctional protein | 0 | 0 | 0 | 2 | 0 | 0 | 0 | 0 | 0 | 0.53% |
| MSBA_PSEAE | msbA | Lipid A export ATP-binding/permease protein | 0 | 0 | 0 | 2 | 0 | 0 | 0 | 0 | 0 | 0.96% |
| NADE_PSEAE | nadE | NH(3)-dependent NAD(+) synthetase | 0 | 0 | 0 | 4 | 2 | 0 | 0 | 0 | 4 | 7.14% |
| NDK_PSEAE | ndk | Cluster of Nucleoside diphosphate kinase | 0 | 5 | 8 | 4 | 0 | 5 | 0 | 0 | 14 | 24.57% |
| NIRF_PSEAE | nirF | Protein NirF | 0 | 0 | 0 | 0 | 0 | 0 | 0 | 0 | 5 | 2.07% |
| NIRS_PSEAE | nirS | Nitrite reductase | 2 | 2 | 4 | 11 | 4 | 13 | 6 | 0 | 24 | 14.63% |
| NOSZ_PSEAE | nosZ | Nitrous-oxide reductase | 0 | 0 | 0 | 8 | 0 | 8 | 3 | 0 | 10 | 5.73% |
| NQOR_PSEAE | PA0949 | NAD(P)H dehydrogenase (quinone) | 0 | 0 | 0 | 0 | 0 | 0 | 0 | 0 | 2 | 1.57% |
| NUOCD_PSEAE | nuoC | NADH-quinone oxidoreductase subunit C/D | 0 | 0 | 0 | 2 | 0 | 0 | 0 | 0 | 3 | 1.67% |
| NUOF_PSEAE | nuoF | NADH-quinone oxidoreductase subunit F | 0 | 0 | 0 | 2 | 0 | 0 | 0 | 0 | 5 | 3.02% |
| NUOG_PSEAE | nuoG | NADH-quinone oxidoreductase subunit G | 0 | 0 | 2 | 6 | 3 | 0 | 0 | 0 | 10 | 4.12% |
| NUSG_PSEAE | nusG | Transcription termination/antitermination protein | 0 | 0 | 2 | 4 | 3 | 2 | 0 | 0 | 4 | 11.99% |
| ODO2_PSEAE | sucB | Dihydrolipoyllysine-residue succinyltransferase component of 2-oxoglutarate dehydrogenase complex | 0 | 4 | 5 | 12 | 6 | 3 | 5 | 4 | 13 | 15.69% |
| ODP1_PSEAE | aceE | Pyruvate dehydrogenase E1 component | 0 | 0 | 3 | 6 | 9 | 2 | 0 | 0 | 7 | 3.90% |
| ODP2_PSEAE | aceF | Dihydrolipoyllysine-residue acetyltransferase component of pyruvate dehydrogenase complex | 0 | 0 | 0 | 7 | 0 | 0 | 0 | 0 | 2 | 1.95% |
| OPGG_PSEAE | opgG | Cluster of Glucans biosynthesis protein G | 0 | 0 | 0 | 0 | 0 | 0 | 0 | 0 | 9 | 3.26% |
| OPGH_PSEAE | opgH | Cluster of Glucans biosynthesis glucosyltransferase H | 0 | 0 | 0 | 7 | 3 | 0 | 0 | 0 | 2 | 2.77% |
| OPRI_PSEAE | oprI | Major outer membrane lipoprotein | 2 | 0 | 0 | 12 | 8 | 0 | 2 | 7 | 7 | 31.58% |
| OTCC_PSEAE | arcB | Cluster of Ornithine carbamoyltransferase, catabolic | 0 | 0 | 0 | 24 | 6 | 4 | 5 | 0 | 24 | 16.48% |
| PAL_PSEAE | pal | Peptidoglycan-associated lipoprotein | 0 | 0 | 0 | 19 | 5 | 3 | 0 | 6 | 14 | 26.44% |
| PCKA_PSEAE | pckA | Cluster of Phosphoenolpyruvate carboxykinase [ATP] | 0 | 0 | 0 | 2 | 3 | 0 | 0 | 0 | 8 | 4.02% |
| PCTA_PSEAE | pctA | Cluster of Methyl-accepting chemotaxis protein | 0 | 3 | 2 | 13 | 12 | 4 | 2 | 2 | 14 | 10.16% |
| PDXH_PSEAE | pdxH | Cluster of Pyridoxine/pyridoxamine 5'-phosphate oxidase | 0 | 0 | 0 | 6 | 3 | 0 | 0 | 0 | 9 | 10.74% |
| PDXJ_PSEAE | pdxJ | Cluster of Pyridoxine 5'-phosphate synthase | 0 | 0 | 0 | 0 | 0 | 0 | 0 | 0 | 3 | 2.96% |
| PGK_PSEAE | pgk | Phosphoglycerate kinase | 0 | 4 | 9 | 5 | 8 | 0 | 0 | 2 | 10 | 14.76% |
| PHEA_PSEAE | pheA | P-protein | 0 | 0 | 0 | 2 | 0 | 0 | 0 | 0 | 3 | 2.35% |
| PHHC_PSEAE | phhC | Aromatic-amino-acid aminotransferase | 0 | 0 | 0 | 3 | 4 | 0 | 0 | 0 | 0 | 3.14% |
| PHS_PSEAE | phhB | Cluster of Pterin-4-alpha-carbinolamine dehydratase | 0 | 2 | 2 | 2 | 2 | 0 | 0 | 0 | 2 | 13.17% |
| PILJ_PSEAE | pilJ | Protein PilJ | 0 | 2 | 0 | 7 | 3 | 0 | 0 | 0 | 12 | 6.22% |
| PILY1_PSEAE | pilY1 | Type IV pilus biogenesis factor | 0 | 0 | 0 | 0 | 0 | 0 | 0 | 0 | 3 | 0.00% |
| PNP_PSEAE | pnp | Cluster of Polyribonucleotide nucleotidyltransferase | 2 | 8 | 11 | 10 | 4 | 18 | 0 | 5 | 14 | 9.93% |
| PORD_PSEAE | oprD | Porin D | 0 | 0 | 0 | 2 | 0 | 0 | 0 | 3 | 0 | 1.30% |
| PORF_PSEAE | oprF | Outer membrane porin F | 0 | 6 | 4 | 20 | 11 | 2 | 4 | 7 | 13 | 21.11% |
| PPIA_PSEAE | ppiA | Peptidyl-prolyl cis-trans isomerase A | 0 | 0 | 0 | 0 | 0 | 0 | 0 | 0 | 4 | 4.22% |
| PPK_PSEAE | ppk | Polyphosphate kinase | 0 | 0 | 0 | 2 | 0 | 0 | 0 | 0 | 0 | 0.47% |
| PROA_PSEAE | proA | Cluster of Gamma-glutamyl phosphate reductase | 0 | 0 | 0 | 2 | 0 | 0 | 0 | 0 | 0 | 0.98% |
| PTA_PSEAE | pta | Phosphate acetyltransferase | 0 | 0 | 0 | 6 | 2 | 0 | 0 | 0 | 0 | 2.30% |
| PUR2_PSEAE | purD | Phosphoribosylamine--glycine ligase | 0 | 0 | 0 | 2 | 2 | 0 | 0 | 0 | 2 | 2.54% |
| PUR4_PSEAE | purL | Cluster of Phosphoribosylformylglycinamidine synthase | 0 | 0 | 5 | 8 | 4 | 0 | 0 | 0 | 4 | 3.68% |
| PUR7_PSEAE | purC | Cluster of Phosphoribosylaminoimidazole-succinocarboxamide synthase | 0 | 0 | 0 | 5 | 0 | 0 | 0 | 0 | 3 | 3.71% |
| PUR8_PSEAE | purB | Adenylosuccinate lyase | 0 | 0 | 0 | 0 | 0 | 0 | 0 | 0 | 4 | 1.37% |
| PUR9_PSEAE | purH | Cluster of Bifunctional purine biosynthesis protein | 0 | 0 | 0 | 2 | 0 | 0 | 0 | 0 | 2 | 1.45% |
| PURA_PSEAE | purA | Cluster of Adenylosuccinate synthetase | 0 | 2 | 6 | 6 | 2 | 2 | 0 | 3 | 12 | 9.51% |
| PURT_PSEAE | purT | Phosphoribosylglycinamide formyltransferase 2 | 0 | 0 | 0 | 0 | 0 | 0 | 0 | 0 | 3 | 1.28% |
| PYRB_PSEAE | pyrB | Cluster of Aspartate carbamoyltransferase | 0 | 0 | 0 | 2 | 0 | 0 | 0 | 0 | 3 | 3.27% |
| PYRC_PSEAE | pyrC | Dihydroorotase | 0 | 0 | 0 | 0 | 0 | 0 | 0 | 0 | 4 | 1.91% |
| PYRE_PSEAE | pyrE | Orotate phosphoribosyltransferase | 0 | 0 | 0 | 0 | 0 | 0 | 0 | 0 | 3 | 2.92% |
| PYRG_PSEAE | pyrG | Cluster of CTP synthase | 0 | 0 | 0 | 2 | 5 | 5 | 0 | 0 | 0 | 1.00% |
| PYRH_PSEAE | pyrH | Uridylate kinase | 0 | 0 | 0 | 0 | 2 | 0 | 0 | 0 | 0 | 1.22% |
| PYRR_PSEAE | pyrR | Bifunctional protein | 0 | 0 | 0 | 0 | 2 | 0 | 0 | 0 | 2 | 3.46% |
| PYRX_PSEAE | pyrC | Dihydroorotase-like protein | 0 | 0 | 0 | 0 | 0 | 0 | 0 | 0 | 3 | 0.89% |
| QUIP_PSEAE | quiP | Acyl-homoserine lactone acylase | 0 | 0 | 0 | 0 | 0 | 0 | 0 | 0 | 2 | 0.35% |
| RAPA_PSEAE | rapA | RNA polymerase-associated protein | 0 | 0 | 0 | 2 | 0 | 0 | 0 | 0 | 0 | 0.40% |
| RBFA_PSEAE | rbfA | Ribosome-binding factor A | 0 | 0 | 0 | 0 | 0 | 0 | 0 | 0 | 2 | 1.98% |
| RECA_PSEAE | recA | Protein RecA | 2 | 0 | 0 | 0 | 2 | 0 | 0 | 0 | 0 | 1.96% |
| RHO_PSEAE | rho | Cluster of Transcription termination factor | 0 | 2 | 2 | 6 | 3 | 0 | 3 | 4 | 5 | 8.93% |
| RIBB_PSEAE | ribB | 3,4-dihydroxy-2-butanone 4-phosphate synthase | 0 | 0 | 0 | 3 | 0 | 0 | 0 | 0 | 2 | 1.92% |
| RISB_PSEAE | ribH | 6,7-dimethyl-8-ribityllumazine synthase | 0 | 0 | 0 | 0 | 0 | 0 | 0 | 0 | 2 | 2.96% |
| RL1_PSEAE | rplA | Cluster of 50S ribosomal protein L1 | 0 | 11 | 8 | 6 | 5 | 0 | 15 | 3 | 19 | 24.42% |
| RL2_PSEAE | rplB | Cluster of 50S ribosomal protein L2 | 0 | 0 | 2 | 12 | 7 | 3 | 2 | 5 | 5 | 18.61% |
| RL3_PSEAE | rplC | Cluster of 50S ribosomal protein L3 | 0 | 0 | 2 | 7 | 4 | 0 | 0 | 3 | 9 | 14.74% |
| RL4_PSEAE | rplD | 50S ribosomal protein L4 | 0 | 2 | 6 | 4 | 4 | 2 | 2 | 0 | 11 | 14.61% |
| RL5_PSEAE | rplE | Cluster of 50S ribosomal protein L5 | 0 | 0 | 4 | 6 | 5 | 0 | 0 | 0 | 9 | 18.50% |
| RL6_PSEAE | rplF | Cluster of 50S ribosomal protein L6 | 0 | 2 | 2 | 6 | 2 | 0 | 0 | 0 | 5 | 11.79% |
| RL7_PSEAE | rplL | Cluster of 50S ribosomal protein L7/L12 | 0 | 2 | 8 | 4 | 4 | 3 | 2 | 0 | 8 | 23.86% |
| RL9_PSEAE | rplI | 50S ribosomal protein L9 | 0 | 6 | 8 | 8 | 7 | 0 | 3 | 4 | 13 | 39.79% |
| RL10_PSEAE | rplJ | Cluster of 50S ribosomal protein L10 | 0 | 3 | 0 | 6 | 9 | 0 | 5 | 4 | 14 | 23.96% |
| RL11_PSEAE | rplK | Cluster of 50S ribosomal protein L11 | 0 | 3 | 6 | 10 | 9 | 0 | 3 | 6 | 11 | 29.07% |
| RL13_PSEAE | rplM | 50S ribosomal protein L13 | 0 | 0 | 4 | 4 | 5 | 0 | 0 | 0 | 12 | 19.33% |
| RL14_PSEAE | rplN | Cluster of 50S ribosomal protein L14 | 0 | 0 | 0 | 0 | 4 | 2 | 4 | 0 | 3 | 3.19% |
| RL15_PSEAE | rplO | 50S ribosomal protein L15 | 0 | 5 | 7 | 4 | 4 | 0 | 2 | 3 | 11 | 27.61% |
| RL17_PSEAE | rplQ | Cluster of 50S ribosomal protein L17 | 0 | 0 | 4 | 8 | 3 | 0 | 2 | 0 | 10 | 18.08% |
| RL18_PSEAE | rplR | Cluster of 50S ribosomal protein L18 | 0 | 3 | 0 | 12 | 15 | 0 | 3 | 8 | 8 | 27.98% |
| RL19_PSEAE | rplS | Cluster of 50S ribosomal protein L19 | 0 | 2 | 3 | 3 | 5 | 0 | 4 | 4 | 6 | 17.61% |
| RL20_PSEAE | rplT | 50S ribosomal protein L20 | 0 | 0 | 0 | 2 | 0 | 0 | 0 | 0 | 0 | 1.79% |
| RL21_PSEAE | rplU | Cluster of 50S ribosomal protein L21 | 0 | 0 | 0 | 0 | 0 | 0 | 0 | 0 | 6 | 6.48% |
| RL22_PSEAE | rplV | Cluster of 50S ribosomal protein L22 | 0 | 0 | 3 | 7 | 0 | 0 | 0 | 2 | 6 | 19.30% |
| RL23_PSEAE | rplW | 50S ribosomal protein L23 | 0 | 0 | 2 | 3 | 3 | 0 | 0 | 0 | 9 | 21.10% |
| RL24_PSEAE | rplX | Cluster of 50S ribosomal protein L24 | 0 | 4 | 4 | 4 | 3 | 0 | 0 | 4 | 10 | 29.49% |
| RL25_PSEAE | rplY | 50S ribosomal protein L25 | 0 | 4 | 5 | 10 | 4 | 0 | 0 | 0 | 9 | 24.72% |
| RL28_PSEAE | rpmB | 50S ribosomal protein L28 | 0 | 2 | 0 | 3 | 2 | 0 | 0 | 0 | 3 | 12.69% |
| RL29_PSEAE | rpmC | Cluster of 50S ribosomal protein L29 | 0 | 3 | 4 | 6 | 5 | 0 | 0 | 3 | 5 | 37.03% |
| RL30_PSEAE | rpmD | 50S ribosomal protein L30 | 0 | 2 | 0 | 4 | 4 | 0 | 0 | 4 | 4 | 24.14% |
| RL31_PSEAE | rpmE | 50S ribosomal protein L31 | 0 | 0 | 2 | 3 | 3 | 0 | 0 | 0 | 0 | 12.99% |
| RL32_PSEAE | rpmF | Cluster of 50S ribosomal protein L32 | 0 | 2 | 6 | 5 | 6 | 2 | 0 | 4 | 6 | 41.11% |
| RL33_PSEAE | rpmG | 50S ribosomal protein L33 | 0 | 0 | 2 | 4 | 3 | 0 | 0 | 0 | 0 | 16.33% |
| RMLC_PSEAE | rmlC | dTDP-4-dehydrorhamnose 3,5-epimerase | 0 | 0 | 0 | 3 | 2 | 0 | 0 | 0 | 0 | 4.54% |
| RNT_PSEAE | rnt | Ribonuclease T | 0 | 0 | 0 | 0 | 2 | 0 | 0 | 0 | 5 | 5.31% |
| RPIA_PSEAE | rpiA | Ribose-5-phosphate isomerase A | 0 | 0 | 0 | 0 | 0 | 0 | 0 | 0 | 4 | 2.79% |
| RPOA_PSEAE | rpoA | Cluster of DNA-directed RNA polymerase subunit alpha | 0 | 10 | 10 | 17 | 14 | 0 | 7 | 9 | 19 | 24.28% |
| RPOC_PSEAE | rpoC | Cluster of DNA-directed RNA polymerase subunit beta | 13 | 12 | 19 | 68 | 51 | 30 | 13 | 10 | 37 | 10.31% |
| RPOD_PSEAE | rpoD | Cluster of RNA polymerase sigma factor | 0 | 0 | 0 | 3 | 0 | 2 | 0 | 0 | 0 | 0.81% |
| RPOZ_PSEAE | rpoZ | DNA-directed RNA polymerase subunit omega | 0 | 0 | 0 | 0 | 3 | 0 | 0 | 0 | 0 | 3.28% |
| RPSH_PSEAE | algU | RNA polymerase sigma-H factor | 0 | 0 | 0 | 2 | 0 | 0 | 0 | 0 | 2 | 3.58% |
| RRAAH_PSEAE | PA1772 | Cluster of Putative 4-hydroxy-4-methyl-2-oxoglutarate aldolase | 0 | 0 | 0 | 0 | 0 | 0 | 0 | 0 | 3 | 1.51% |
| RRF_PSEAE | frr | Ribosome-recycling factor | 0 | 0 | 0 | 0 | 0 | 0 | 0 | 0 | 7 | 3.42% |
| RS1_PSEAE | rpsA | Cluster of 30S ribosomal protein S1 | 3 | 15 | 32 | 29 | 17 | 11 | 14 | 11 | 35 | 34.82% |
| RS2_PSEAE | rpsB | Cluster of 30S ribosomal protein S2 | 0 | 6 | 5 | 12 | 7 | 8 | 0 | 2 | 10 | 18.47% |
| RS3_PSEAE | rpsC | Cluster of 30S ribosomal protein S3 | 0 | 0 | 0 | 2 | 0 | 2 | 0 | 0 | 0 | 2.72% |
| RS4_PSEAE | rpsD | Cluster of 30S ribosomal protein S4 | 0 | 0 | 7 | 6 | 8 | 2 | 2 | 0 | 0 | 11.76% |
| RS5_PSEAE | rpsE | Cluster of 30S ribosomal protein S5 | 0 | 0 | 0 | 8 | 6 | 0 | 2 | 3 | 0 | 14.92% |
| RS6_PSEAE | rpsF | Cluster of 30S ribosomal protein S6 | 3 | 5 | 12 | 13 | 9 | 5 | 4 | 4 | 13 | 49.72% |
| RS7_PSEAE | rpsG | Cluster of 30S ribosomal protein S7 | 0 | 13 | 16 | 11 | 6 | 3 | 12 | 2 | 15 | 43.67% |
| RS8_PSEAE | rpsH | 30S ribosomal protein S8 | 0 | 3 | 3 | 3 | 6 | 2 | 3 | 3 | 7 | 27.09% |
| RS9_PSEAE | rpsI | Cluster of 30S ribosomal protein S9 | 0 | 4 | 2 | 4 | 6 | 0 | 0 | 4 | 4 | 12.83% |
| RS10_PSEAE | rpsJ | Cluster of 30S ribosomal protein S10 | 0 | 0 | 7 | 9 | 5 | 2 | 6 | 3 | 7 | 36.88% |
| RS11_PSEAE | rpsK | Cluster of 30S ribosomal protein S11 | 0 | 0 | 2 | 4 | 3 | 0 | 0 | 2 | 3 | 13.97% |
| RS12_PSEAE | rpsL | Cluster of 30S ribosomal protein S12 | 0 | 0 | 0 | 4 | 6 | 0 | 3 | 5 | 3 | 8.32% |
| RS13_PSEAE | rpsM | Cluster of 30S ribosomal protein S13 | 0 | 0 | 2 | 0 | 2 | 2 | 0 | 0 | 0 | 7.44% |
| RS14_PSEAE | rpsN | 30S ribosomal protein S14 | 0 | 0 | 0 | 2 | 0 | 0 | 0 | 0 | 3 | 9.57% |
| RS15_PSEAE | rpsO | 30S ribosomal protein S15 | 0 | 0 | 0 | 0 | 0 | 0 | 0 | 0 | 4 | 6.24% |
| RS16_PSEAE | rpsP | 30S ribosomal protein S16 | 0 | 0 | 0 | 3 | 3 | 0 | 0 | 0 | 2 | 14.86% |
| RS17_PSEAE | rpsQ | Cluster of 30S ribosomal protein S17 | 0 | 0 | 0 | 5 | 8 | 2 | 0 | 5 | 7 | 31.32% |
| RS18_PSEAE | rpsR | 30S ribosomal protein S18 | 0 | 0 | 0 | 3 | 3 | 0 | 0 | 0 | 3 | 16.37% |
| RS19_PSEAE | rpsS | Cluster of 30S ribosomal protein S19 | 0 | 2 | 6 | 10 | 9 | 0 | 8 | 0 | 9 | 22.22% |
| RS20_PSEAE | rpsT | Cluster of 30S ribosomal protein S20 | 0 | 0 | 2 | 2 | 0 | 2 | 0 | 0 | 6 | 13.80% |
| RS21_PSEAE | rpsU | Cluster of 30S ribosomal protein S21 | 0 | 5 | 8 | 7 | 6 | 0 | 3 | 0 | 7 | 29.88% |
| SAHH_PSEAE | ahcY | Cluster of Adenosylhomocysteinase | 0 | 0 | 0 | 3 | 0 | 0 | 0 | 0 | 3 | 2.46% |
| SECA_PSEAE | secA | Cluster of Protein translocase subunit | 3 | 0 | 0 | 4 | 4 | 7 | 0 | 0 | 2 | 2.41% |
| SECB_PSEAE | secB | Protein-export protein | 0 | 0 | 0 | 0 | 0 | 0 | 0 | 0 | 5 | 1.91% |
| SECD_PSEAE | secD | Protein translocase subunit | 0 | 0 | 0 | 18 | 9 | 0 | 2 | 4 | 14 | 11.71% |
| SECF_PSEAE | secF | Protein translocase subunit | 0 | 0 | 0 | 0 | 0 | 0 | 0 | 0 | 2 | 0.91% |
| SECG_PSEAE | secG | Protein-export membrane protein | 0 | 0 | 0 | 2 | 0 | 0 | 0 | 0 | 0 | 2.84% |
| SECY_PSEAE | secY | Protein translocase subunit | 0 | 0 | 0 | 2 | 0 | 0 | 0 | 0 | 0 | 0.60% |
| SERC_PSEAE | serC | Phosphoserine aminotransferase | 0 | 0 | 0 | 0 | 2 | 0 | 0 | 0 | 0 | 1.05% |
| SKPL_PSEAE | PA3647 | Skp-like protein | 0 | 0 | 0 | 3 | 0 | 0 | 0 | 0 | 9 | 8.87% |
| SODF_PSEAE | sodB | Superoxide dismutase [Fe] | 0 | 0 | 2 | 0 | 0 | 0 | 0 | 0 | 0 | 2.88% |
| SPUE_PSEAE | spuE | Spermidine-binding periplasmic protein | 0 | 0 | 0 | 0 | 0 | 0 | 0 | 2 | 0 | 1.10% |
| SSB_PSEAE | ssb | Single-stranded DNA-binding protein | 0 | 2 | 0 | 0 | 2 | 0 | 0 | 0 | 4 | 5.87% |
| SUCC_PSEAE | sucC | Cluster of Succinyl-CoA ligase [ADP-forming] subunit beta | 2 | 2 | 2 | 15 | 13 | 2 | 0 | 4 | 7 | 17.64% |
| SUCD_PSEAE | sucD | Cluster of Succinyl-CoA ligase [ADP-forming] subunit alpha | 0 | 0 | 0 | 8 | 7 | 0 | 0 | 2 | 4 | 11.83% |
| SUHB_PSEAE | suhB | Inositol-1-monophosphatase | 0 | 0 | 0 | 0 | 0 | 0 | 0 | 0 | 3 | 1.56% |
| SURA_PSEAE | surA | Chaperone | 0 | 0 | 0 | 3 | 2 | 0 | 0 | 0 | 6 | 5.27% |
| SYA_PSEAE | alaS | Cluster of Alanine--tRNA ligase | 0 | 0 | 0 | 3 | 3 | 0 | 0 | 0 | 2 | 1.39% |
| SYDND_PSEAE | aspS | Cluster of Aspartate--tRNA(Asp/Asn) ligase | 2 | 0 | 2 | 0 | 5 | 3 | 0 | 0 | 0 | 2.11% |
| SYFB_PSEAE | pheT | Phenylalanine--tRNA ligase beta subunit | 0 | 0 | 0 | 2 | 0 | 0 | 0 | 0 | 2 | 1.01% |
| SYGB_PSEAE | glyS | Glycine--tRNA ligase beta subunit | 0 | 0 | 0 | 2 | 0 | 0 | 0 | 0 | 0 | 0.44% |
| SYI_PSEAE | ileS | Cluster of Isoleucine--tRNA ligase | 0 | 0 | 0 | 2 | 0 | 0 | 0 | 0 | 0 | 0.35% |
| SYK_PSEAE | lysS | Cluster of Lysine--tRNA ligase | 0 | 0 | 4 | 5 | 4 | 0 | 0 | 0 | 12 | 7.66% |
| SYM_PSEAE | metG | Methionine--tRNA ligase | 0 | 0 | 0 | 0 | 0 | 2 | 0 | 0 | 0 | 0.44% |
| SYP_PSEAE | proS | Cluster of Proline--tRNA ligase | 0 | 2 | 2 | 4 | 2 | 0 | 2 | 0 | 14 | 6.47% |
| SYQ_PSEAE | glnS | Cluster of Glutamine--tRNA ligase | 0 | 0 | 0 | 2 | 3 | 0 | 0 | 0 | 0 | 1.24% |
| SYR_PSEAE | argS | Cluster of Arginine--tRNA ligase | 0 | 0 | 0 | 4 | 4 | 0 | 0 | 0 | 0 | 1.40% |
| SYW_PSEAE | trpS | Tryptophan--tRNA ligase | 0 | 0 | 0 | 0 | 0 | 0 | 0 | 0 | 3 | 1.21% |
| SYY2_PSEAE | tyrS2 | Tyrosine--tRNA ligase 2 | 0 | 0 | 2 | 5 | 0 | 2 | 0 | 0 | 8 | 8.00% |
| TAL_PSEAE | tal | Cluster of Transaldolase | 0 | 0 | 0 | 0 | 2 | 0 | 0 | 0 | 0 | 1.01% |
| TATA_PSEAE | tatA | Sec-independent protein translocase protein | 0 | 0 | 0 | 3 | 0 | 0 | 0 | 0 | 5 | 9.08% |
| THIG_PSEAE | thiG | Thiazole synthase | 0 | 0 | 0 | 0 | 0 | 0 | 0 | 0 | 2 | 1.01% |
| THIO_PSEAE | trxA | Thioredoxin | 0 | 3 | 8 | 6 | 6 | 0 | 2 | 2 | 10 | 40.13% |
| THRC_PSEAE | thrC | Threonine synthase=3 SV=3 | 0 | 0 | 0 | 2 | 0 | 0 | 0 | 0 | 0 | 0.62% |
| TIG_PSEAE | tig | Cluster of Trigger factor | 0 | 5 | 10 | 12 | 11 | 5 | 2 | 5 | 13 | 23.62% |
| TOLQ_PSEAE | tolQ | Protein TolQ | 0 | 0 | 3 | 5 | 2 | 0 | 0 | 2 | 5 | 10.97% |
| TPIS_PSEAE | tpiA | Triosephosphate isomerase | 0 | 0 | 0 | 0 | 3 | 0 | 0 | 0 | 0 | 1.72% |
| TRPC_PSEAE | trpC | Cluster of Indole-3-glycerol phosphate synthase | 0 | 0 | 0 | 0 | 0 | 0 | 0 | 0 | 2 | 0.88% |
| UBIE_PSEAE | ubiE | Cluster of Ubiquinone/menaquinone biosynthesis C-methyltransferase | 0 | 0 | 0 | 2 | 2 | 0 | 0 | 0 | 0 | 2.47% |
| UBIG_PSEAE | ubiG | Ubiquinone biosynthesis O-methyltransferase | 0 | 0 | 0 | 4 | 0 | 0 | 0 | 0 | 2 | 5.08% |
| UGND_PSEAE | wbpA | UDP-N-acetyl-D-glucosamine 6-dehydrogenase | 0 | 0 | 4 | 7 | 5 | 0 | 0 | 0 | 9 | 10.03% |
| UPP_PSEAE | upp | Cluster of Uracil phosphoribosyltransferase | 0 | 2 | 0 | 0 | 3 | 0 | 0 | 0 | 4 | 5.13% |
| WBPE_PSEAE | wbpE | UDP-2-acetamido-2-deoxy-3-oxo-D-glucuronate aminotransferase | 0 | 0 | 4 | 3 | 2 | 0 | 0 | 2 | 4 | 6.35% |
| WBPI_PSEAE | wbpI | UDP-2,3-diacetamido-2,3-dideoxy-D-glucuronate 2-epimerase | 0 | 0 | 0 | 3 | 3 | 0 | 0 | 2 | 0 | 3.22% |
| Y329_PSEAE | PA0329 | UPF0339 protein PA0329 | 0 | 0 | 0 | 0 | 2 | 0 | 0 | 0 | 3 | 8.49% |
| Y423_PSEAE | PA0423 | UPF0312 protein PA0423 | 0 | 2 | 2 | 0 | 0 | 0 | 0 | 0 | 6 | 12.34% |
| Y1574_PSEAE | PA1574 | UPF0345 protein PA1574 | 0 | 0 | 2 | 0 | 0 | 0 | 0 | 0 | 0 | 2.99% |
| Y2980_PSEAE | PA2980 | UPF0434 protein PA2980 | 0 | 0 | 0 | 2 | 0 | 0 | 0 | 0 | 0 | 5.64% |
| Y3922_PSEAE | PA3922 | Uncharacterized protein PA3922 | 0 | 0 | 0 | 0 | 0 | 0 | 0 | 3 | 0 | 1.24% |
| Y4473_PSEAE | PA4473 | UPF0307 protein PA4473 | 0 | 0 | 2 | 0 | 0 | 0 | 0 | 0 | 2 | 4.28% |
| Y4489_PSEAE | PA4489 | UPF0192 protein PA4489 | 0 | 0 | 2 | 7 | 7 | 0 | 0 | 3 | 9 | 3.20% |
| Y4667_PSEAE | PA4667 | TPR repeat-containing protein PA4667 | 0 | 0 | 0 | 0 | 0 | 0 | 0 | 0 | 6 | 1.82% |
| Y4738_PSEAE | PA4738 | UPF0337 protein PA4738 | 0 | 0 | 0 | 0 | 0 | 2 | 0 | 0 | 0 | 4.44% |
| Y4753_PSEAE | PA4753 | Probable RNA-binding protein PA4753 | 0 | 0 | 0 | 0 | 0 | 0 | 0 | 0 | 2 | 2.36% |
| Y4798_PSEAE | PA4798 | UPF0157 protein PA4798 | 0 | 0 | 0 | 0 | 0 | 0 | 0 | 0 | 2 | 1.88% |
| YIDC_PSEAE | yidC | Membrane protein insertase | 0 | 0 | 0 | 6 | 4 | 0 | 0 | 2 | 4 | 5.77% |
| ZIPA_PSEAE | zipA | Cluster of Cell division protein | 0 | 0 | 0 | 5 | 0 | 2 | 0 | 2 | 7 | 11.14% |

1. [↑](#footnote-ref-1)
